# Patients with Schizophrenia and Social Contacts

**DOI:** 10.3889/oamjms.2016.084

**Published:** 2016-07-21

**Authors:** Slavica Arsova, Gabriela Kopacheva Barsova

**Affiliations:** 1 *University Psychiatry Clinic, Faculty of Medicine, Ss Cyril and Methodius University of Skopje, Skopje, Republic of Macedonia*; 2 *University Clinic for Ear, Nose and Throat, Faculty of Medicine, Ss Cyril and Methodius University of Skopje, Vodnjanska 17, Skopje 1109, Republic of Macedonia*

**Keywords:** schizophrenia, treatment, personal and social relations, psychosocial activity, Republic of Macedonia

## Abstract

**BACKGROUND:**

Patients with schizophrenia have severe problems with personal and social relations which affect their quality of life.

**AIM:**

The aim of the paper was to monitor personal and social relations in patients with schizophrenia and to find out the differences regarding socio-demographic characteristics and ambulatory and day hospital treatment.

**MATERIAL AND METHODS:**

The investigation included 120 subjects each with diagnosis F20 according to ICD 10 criteria; divided into two groups of 60 patients regarding their actual treatment (the first group received ambulatory care whereas those from the second group had a day hospital treatment). Patients were of different age and gender, receiving regular antipsychotic therapy. They were included in individual and group psychosocial therapeutic procedures during the day hospital treatment. The investigation utilised the following diagnostic instruments: standardised clinical interview and Personal and social performance scale (PSP scale), a non-standardized questionnaire of socio-demographic data, family support and existence of mental disorder in other family members.

**RESULTS:**

The results have shown better personal and social functioning in patients who had family support, in those who are employed, in those with no mental disorder in other family members and in patients on day hospital treatment against patients receiving ambulatory care.

**CONCLUSION:**

Day hospital treatment, family support and social support improve the ability for personal and social contacts of patients with schizophrenia.

## Introduction

Over the last two decades, psychosocial activities have been directed towards to improve the personal and social functioning of patients with schizophrenia which means not only treatment of the schizophrenic symptomatology. Schizophrenia is a chronic mental disorder that affects emotions, cognition behaviour. The consequences are poor psychosocial functioning and a low quality of life in those people.

The quality of life means the ability to play socially defined roles such as homemaker, worker, student, spouse and friend, and additionally, this gives the individual a feeling of satisfaction and the ability to take care of him/her and to enjoy the life [1].

Psychosocial interventions inducted on a day hospital treatment would enable better therapeutic collaboration, effective pharmacological treatment, better control of patient disorder and their life in general and taking self-care of themselves with greater personal satisfaction [2-4].

The aim of this study was to monitor self-care in patients with schizophrenia and to find out the differences regarding socio-demographic characteristics and ambulatory and day hospital treatment.

## Materials and Method

The investigation included 120 subjects each with diagnosis F20 according to ICD 10 criteria. Subjects were divided into two groups of 60 patients regarding their actual treatment. The first group received ambulatory care whereas those from the second group had a day hospital treatment). Patients were of different age and gender and were, receiving regular antipsychotic therapy. They were included in individual and group psychosocial therapeutic procedures during the day hospital treatment. The subjects of both groups were evaluated at the beginning of treatment and after 6 months, after ambulatory or day hospital treatment.

The investigation utilised the following diagnostic instruments: standardised clinical interview; personal and social performance scale (PSP scale) [5]; non-standardized questionnaire of socio-demographic data including, family support and existence of mental disorder in other family members.

## Results

Distribution in Table 1 shows the absence of problems in personal and social contacts in only 4 (3.33%) single subjects, but severe problems were found in 18 (15%) of subjects and very severe in 32 (26.67%) of single subjects. The final results of the research have shown that the majority of the subjects who are with schizophrenia are singles not married with statistical signification p = 0.017.

**Table 1:** Personal and social contacts – marital status

Personal and social contacts	Marital status				*Total*
	Single/man/woman	Married	Divorced	Widow	
1 absent	4 (3.33%)	2 (1.67%)	1 (0.83%)	0	7 (5.83%)
2 mild	4 (3.33%)	1 (0.83%)	2 (1.67%)	0	7 (5.83%)
3 manifested	4 (3.33%)	6 (5.0%)	0	0	10 (8.33%)
4 marked	12 (10.0%)	14 (11.67%)	1 (0.83%)	1 (0.83%)	28 (23.33%)
5 severe	18 (15.0%)	5 (4.17%)	2 (1.67%)	0	25 (20.83%)
6 very severe	32 (26.67%)	6 (5.0%)	4 (3.33%)	1 (0.83%)	43 (35.83%)
Total	74 (61.67%)	34 (28.33%)	10 (8.33%)	2 (1.67%)	120 (100%)

Kruskal-Wallis H = 8.13, p = 0.017.

**Table 2:** Personal and social contacts – educational level

Personal and social contacts	Education			Total
	Low	High	Academic level	
1 absent	0	5 (4.17%)	2 (1.67%)	7 (5.83%)
2 mild	0	1 (0.83%)	6 (5.0%)	7 (5.83%)
3 manifested	1 (0.83%)	5 (4.17%)	4 (3.33%)	10 (8.33%)
4 marked	2 (1.67%)	19 (15.83%)	7 (5.83%)	28 (23.33%)
5 severe	3 (2.50%)	17 (14.17%)	5 (4.17%)	25 (20.83%)
6 very severe	11 (9.17%)	25 (20.83%)	7 (5.83%)	43 (35.83%)
Total	17 (14.17%)	72 (60.0%)	31 (25.83%)	120 (100%)

Kruskal-Wallis H = 11.99, p = 0.0025.

The subjects who have the lower education or they are not employed have significantly harder tasks in establishing of personal and social contacts versus the ones who are employed and possessing a higher level of education, p = 0.0025.

Distribution in Table 3 shows the absence of problems in personal and social contacts in 3 (2.50) unemployed, 1 (0.83%) employed subjects and in 2 (1.67%) retired persons. Manifested problems were found in 6 (5.0%) unemployed, 4 (3.33) employed subjects, 1 student and 2 retired subjects, whereas marked problems were found in 15 (12.50%) unemployed subjects. Very severe problems in social relations were experienced by 34 (28.33%) unemployed subjects.

**Table 3:** Personal and social contacts – employment status of the subjects

Personal and social contacts	Employment				Total
	Unemployed	Employed	Student	Retired persons	
1 absent	3 (2.50%)	1 (0.83%)	1 (0.83%)	2 (1.67%)	7 (5.83%)
2 mild	3 (2.50%)	1 (0.83%)	0	3 (2.50%)	7 (5.83%)
3 manifested	6 (5.0%)	4 (3.33%)	0	0	10 (8.33%)
4 marked	15 (12.50%)	13 (10.83%)	0	0	28 (23.33%)
5 severe	20 (16.67%)	1 (0.83%)	0	4 (3.33%)	25 (20.83%)
6 very severe	34 (28.33%)	4 (3.33%)	1 (0.83%)	4 (3.33%)	43 (35.83%)
Total	81 (67.50%)	24 (20.0%)	2 (1.67%)	13 (10.83%)	120 (100%)

Kruskal-Wallis H=7.13, p=0.028.

**Table 4:** Personal and social contacts – family support

Self-care	I think the family is supportive							Total
		No		Little		Very much		
1 absent	1	(0.83%)	4	(3.33%)	2	(1.67%)	7	(5.83%)
2 mild	1	(0.83%)	1	(0.83%)	5	(4.17%)	7	(5.83%)
3 manifested	1	(0.83%)	3	(2.50%)	6	(5.0%)	10	(8.33%)
4 marked	1	(0.83%)	9	(7.50%)	18	(15.0%)	28	(23.33%)
5 severe	2	(1.67%)	17	(14.17%)	6	(5.0%)	25	(20.83%)
6 very severe	11	(9.17%)	22	(18.33%)	10	(8.33%)	43	(35.83%)
Total	17	(14.17%)	56	(46.67%)	47	(39.17%)	120	(100%)

Chi-square = 45.65, df = 10, p = 0.000000.

**Table 5:** Personal and social contacts – DC/Ambulatory Care

Personaland social contacts / 6 M	DC			Ambulatory Care
	N	%	N	%
1 absent	6	10.0	2	3.33
2 mild	34	56.67	5	8.33
3 manifest	16	26.67	20	33.33
4 marked	3	5.0	17	28.33
5 severe	1	1.67	14	23.33
6 very severe	0	0	2	3.33
Total	60	100	60	100

U = 588.5, Z = 6.36, P = 0.00000.

Subjects with different employment status (p<0.05) showed differences in the modalities of the personal and social contacts Scale. Unemployed subjects significantly more often had marked or severe problems regarding the personal and social relations, while employed subjects significantly less often had very severe problems.

Six months after the grouping of subjects, in the day hospital treatment group had - mild difficulties of self-care [34 (56.7%)], with the manifestation of problems in 16 (26.7%). In ambulatory treated patients 20 (33.3%) demonstrated manifest problems; 17 (28.3%) had marked problems and-14 (23.3%), severe difficulties in taking care of themselves. There was a high statistically significant difference between subjects who were treated daily in a hospital and those who were ambulatory-cared. Patients who were treated on a daily basis in the hospital did not have any hard times regarding their daily self-care (p<0.0001).

## Discussion

The results obtained in this study demonstrated unsatisfactory psychosocial functioning in both groups of patients. Thus, quality of life of patients with the schizophrenic disorder was observed. Most of the unemployed patients, about 30% lost their jobs after because of the psychosocial dysfunction and stigmatisation of the society.

However, the six–month continuous treatment brought improvement in functioning, which was statistically significant in those receiving day hospital treatment. Our results are in therefore in agreement with those presented by other authors, who suggested that integrated psychopharmacological and psychosocial treatment was indispensable for the inclusion of these patients again in the social functioning, establishing social contacts, employment, as part of, inclusion in societal life [1, 2, 4, 6, 8, 9].

NICE rec. for the treatment and recovery of the patients with schizophrenia is a community-based treatment which means individual treatment tailored for each patient, treatment in the community, ambulatory care, service level interventions, and acute day hospital treatment and in the day hospital centres. NICE rec. are CBT treatment, family interventions and art therapy in the recovery period and after for faster and better reintegration and socialisation [21].

Data presented in literature point out to the poor psychosocial functioning of patients with schizophrenic disorders and poor quality of life in general [9]. Koivumaa-Honkanen et al. in their investigation used different scales for assessment of the quality of life (QOL) in patients with schizophrenia and found out poorer functioning in these patients compared to the remaining psychiatric patients [10]. Sullivan et al. conducted a study among a population of schizophrenic patients divided into three groups (patients in psychiatric institutions, patients who live alone and those who live in centres for psychosocial support) and compared them with the healthy population. Using the interview for the assessment of QOL, they obtained results that revealed the poorer quality of life in all three groups of schizophrenic subjects against the healthy ones. The biggest differences were observed in satisfaction from social life, finances and employment.

Malm et al. using the semi-structured questionnaire (QOLC) for assessment of the quality of life of 40 schizophrenic subjects 2 years after their last hospitalisation, found out dissatisfaction in almost all aspects of living and especially in social relations, education, finances, etc. [11].

The majority of studies identify the relationship and diversity of quality of life in schizophrenic patients and some sociodemographic characteristics [12]. Shtasel et al. in their study of schizophrenic patients detected better functioning of female subjects than male [13, 14].

On the other hand, Lehman in his study revealed that individuals who were married had a better quality of life that those who were not married [15, 16]. With regard to education, many studies have revealed the poorer quality of life in those schizophrenic patients who had higher levels of education [17]. Other researchers have presented the correlation between the presence of neuroleptic symptomatology, negative schizophrenic symptomatology and distinct depression with low quality of life satisfaction [17, 18].

Relationship between the treatment of these patients and their quality of life is underlined in many studies the results obtained confirmed better psychosocial functioning with usage of the second-generation antipsychotics and, better quality of life in those subjects who had integrated psychopharmacological and psychosocial treatment (family interventions, supportive interventions, cognitive behavioural, training for social skills, and especially in day hospital settings or other similar psychosocial facilities) [1, 3, 19, 20].

In conclusion, daily hospital psychosocial therapeutic treatment in combination with regular antipsychotic therapy, family and social support helps in more rapid reintegration and re-socialization and better quality of life in patients with schizophrenia.
